# Involvement of bilateral insula in brand extension evaluation: an fMRI study

**DOI:** 10.1038/s41598-021-83057-8

**Published:** 2021-02-09

**Authors:** Taeyang Yang, Ji-Hyun Kim, Junsuk Kim, Sung-Phil Kim

**Affiliations:** 1 Department of Biomedical Engineering, Ulsan National Institute of Science and Technology, UNIST-Gil 50, Ulsan, 44919 Republic of Korea; 2 Department of Industrial ICT Engineering, Dong-Eui University, Eomgwangno 176, Busan, 47340 Republic of Korea

**Keywords:** Functional magnetic resonance imaging, Decision, Human behaviour

## Abstract

The present study aims to investigate functional involvement of brain areas in consumers’ evaluation of brand extension that refers to the use of well-established brand for launching new offerings. During functional magnetic resonance imaging (fMRI) scanning, participants viewed a beverage brand name followed by an extension goods name selected from the beverage or household appliance categories. They responded acceptability to given brand extension. Both acceptability responses and reaction time revealed a noticeable pattern that participants responded to acceptable stimuli more carefully. General linear model (GLM) analyses revealed the involvement of insular activity in brand extension evaluation. Especially, insular activity was lateralized according to valence. Furthermore, its activity could explain behavioral response in parametric modulation model. According to these results, we speculate that insula activity is relevant to emotional processing. Finally, we divided neural activities during brand extension into separated clusters using a hierarchical clustering-based connectivity analysis. Excluding two of them related to sensorimotor functions for behavioral responses, the remaining cluster, including bilateral insula, was likely to reflect brand extension assessment. Hence, we speculate that consumers’ brand extension evaluation may involve emotional processes, shown as insular activity.

## Introduction

Brand extension refers to the use of well-established brand name for promoting the new service or goods^1^. Brand extension provides a way to take advantage of well-established brand name recognition and image to enter new markets, reducing the risk for failure and the cost for promotion^2^. However, the failure of brand extension strategy (i.e. poor fits between parent brand and extended goods) can also lead to negative perceptions, so called brand name dilution^3^, stimulating undesirable beliefs and association in consumers’ mind^2^. Therefore, how consumers evaluate brand extension has posed an important question for marketing research.

Traditionally, researchers have addressed this question using explicit evaluation techniques, such as surveys or focus group interviews^4^. The previous results indicate that greater similarity between new goods and parent brand (category) increases the likelihood of successful brand extension^2^. However, explicit techniques have limitations in investigating endogenous processes, so it remains questionable what cognitive processes are engaged in the evaluation of brand extension.

With the recent advances in cognitive neuroscience, researchers have attempted to investigate cognitive processes underlying brand extension evaluation through the analysis of consumers’ brain activity. For example, Ma, et al.^5^ investigated consumers’ responses to brand extension by analyzing electroencephalography (EEG), revealing a positive relationship between event-related potential (ERP)’s (e.g. N270) and the level of conflicts between brand and extension categories. Subsequently, Ma et al.^4^ suggested that the similarity-based categorization process is essential to decision making in brand extension evaluation. Later, Ma et al.^6^ proposed the two-stage process underlying brand extension evaluation, which includes an early low-level similarity-based process followed by a late high-level category-based process. Recently, other studies have reported the effect of culture-dependent consumers’ attitude^7^, extension stimulus type^8^ or offering type^9,10^ on brand extension evaluation.

Nevertheless, these previous studies have investigated brain activity using only scalp EEG. Although EEG is a useful neurophysiological apparatus with a good temporal resolution, it intrinsically has a poor spatial resolution and carries a limited amount of neural information. These drawbacks make it difficult to employ EEG measurements to directly examine a wide range of brain regions including in-depth brain structures. Many in-depth regions are known to be involved in diverse cognitive/affective processes for emotion, fear, or memory. Gardner and Levy^11^ defined a “brand” as a complex symbol representing a variety of ideas and attributes, which cannot be described in a simple text^12^. Therefore, it would be a valid assumption that not only cortical but also other in-depth brain regions are involved in brand extension evaluation. In this regard, EEG-based inferences of underlying cognitive processes may not be sufficient to broaden our understanding of consumers’ evaluation of brand extension. To overcome this limitation, functional magnetic resonance imaging (fMRI) can offer an alternative means to measure in-depth as well as cortical activities with a fine spatial resolution.

Therefore, the goal of this study is to investigate how in-depth brain activities are modulated during brand extension evaluation using fMRI and what cognitive and affective processes they reflect. To this end, we employed a goods-to-goods brand extension scenario in our experiment and designed a brand extension evaluation paradigm in which we serially presented a pair of brand (S1) and extended goods (S2) stimuli followed by evaluation responses. This brand extension evaluation paradigm was adjusted to enable human experiments in the MRI scanner so that we could obtain brain signals concurrently with behavioral evaluation. We then analyzed brain activities over the whole brain fMRI data relative to behavioral responses. Specifically, we identified brain activities that distinguished between high-, and low-fit brand extension, which were determined based on not the stimulus design prior to experiment, but actual acceptability values provided by study participants during experiment. Finally, we conducted hierarchical clustering analysis to partition brain activities during brand extension evaluation and compared functional connectivity according to participant’s evaluation.

## Methods

Thirty healthy participants (9 Female) with a mean age of 22.60 years old (SE 0.37, range 18–27 years old) participated in the study on the basis of informed consent. The study was conducted according to the Declaration of Helsinki, with the approval of UNIST IRB committee (UNISTIRB-19-04-C). All participants had normal or corrected-to-normal vision and normal hearing. No participant had a history of neurological, major medical, or psychiatric disorder. Three participants’ data were excluded in the analysis due to excessive head movements over the voxel size (i.e., 2 mm).

**Figure 1:**
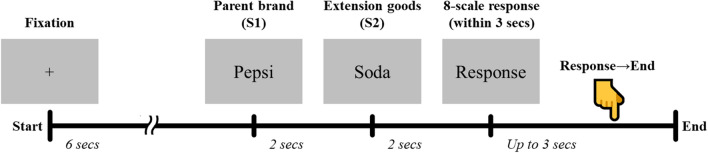
Experimental design (S1–S2 paradigm). Each trial began with the fixation point of 6 s. In next, parent brand name (S1) and extension goods name (S2) were presented successively for 2 s each. Participants were then instructed to evaluate brand extension pair with 8-scale response by both hands within 3 s. The trial ended when either participant responded within 3 s or fixed time (i.e., 3 s) is over.

Participants were provided goods-to-goods brand extension samples and asked to evaluate each sample during fMRI scanning (Fig. 1). The S1–S2 paradigm, which has been widely used in the previous EEG studies^4,5,9,10^, was modified here to fit to fMRI scanning. In the beginning of each trial, a white cross appeared at the center of the black screen for 6 s. Then, a parent brand name (S1) in white characters was presented at the center of the black screen for 2 s, followed by the extension goods name (S2) for another 2 s. When S2 disappeared, the word “Response” appeared on the screen to inform participants to respond how acceptable the presented pair was in a 8-scale measure (i.e., − 4, − 3, − 2, − 1, 1, 2, 3, and 4). Participants responded by pressing one of the eight buttons (i.e., D5_L_ (the fifth digit of the left hand), D4_L_, D3_L_, and D2_L_ for − 4, − 3, − 2, and − 1, and D2_R_ (the second digit of the right hand), D3_R_, D4_R_, and D5_R_ for 1, 2, 3, and 4). Note that we did not include 0 in the response scale to avoid an ambiguous answer. There were 53 trials with missing responses across 27 participants (1.96 ± 1.40 trial per participant). Therefore, we excluded those trials from all the subsequent analysis procedures, including general linear model (GLM) and clustering analyses as well as behavioral analysis.

To create brand extension samples, we conducted a pilot study similar to the one^7^. Fifty Korean participants not involved in the main study were asked to write beverage brand names familiar to themselves for 7 min. After gathering data, we chose top-ranked 6 brand names except for those with an competitive brand (e.g. Coke and Pepsi) as follows: Pepsi (cola), Chilsung cider (soda), Pocari sweat (sports drink), Seoul milk (milk), Del monte (juice), and Minute Maid (juice). Next, we chose 5 beverage goods names (used for beverage-to-beverage (*BB*) brand extension) and 5 household appliance names (used for beverage-to-household (*BH*) brand extension), which have been used in the previous EEG studies^4–6,13^. These 10 goods names (S2) were fully combined with 6 brand names (S1), creating 60 S1–S2 pairs. Sixty S1–S2 brand extension pairs were presented in three experimental sessions (i.e., three repetition), resulting in total 180 trials per participant.

The MRI scanning was conducted using a 3 T scanner (Magnetom TrioTim, Siemens, Germany) with a 64-channel head coil at the Center for Neuroscience Imaging Research (CNIR) in Suwon, Republic of Korea. 3-D functional images were acquired using a slice-accelerated multiband gradient-echo-based echo planar imaging (EPI) sequence using T2*-weighted blood oxygenation level dependent (BOLD) contrast, covering the whole brain region (multiband acceleration factor = 2, number of slices = 72, repetition time (TR) = 2000 ms, echo time (TE) = 35 ms, flip angle = 90°, Field of view (FOV) = 200 mm, slice thickness = 2 mm and voxel size = 2.0 × 2.0 × 2.0 mm^3^). Also, anatomical high-resolution images were obtained (T1-weighted 3D MPRAGE sequence, TR = 2300 ms, TE = 2.28 ms, flip angle = 8°, FOV = 256 mm, and voxel size = 1.0 × 1.0 × 1.0 mm^3^).

To analyze behavioral data, we firstly verified our prior conjecture that participants would positively respond to *BB* and negatively respond to *BH* brand extension pairs in general, by calculating the mean responses of each type of brand extension pair (Fig. 3). According to the result (see “Results”), we grouped the stimuli based on the behavioral responses (i.e., positive vs negative), not on the pre-determined design (i.e., *BB* vs *BH*), and compared brain signals between the two groups. Also, we compared behavioral results [i.e., absolute value of responses and reaction time (RT)] between the positive and negative response groups with a paired t-test. As a last behavioral analysis, we compared all participants’ response by aggregating samples.

The fMRI data were analyzed using SPM12 software (Wellcome Department of Imaging Neuroscience, London, UK). We performed event-related fMRI analyses of participants’ BOLD signals. Using the SPM software, functional Echo Planar Imaging (EPI) images were preprocessed for slice timing and realignment to the first volume of each run. The participants were excluded if the head movement exceeded the size of voxel (i.e., 2 mm). Then, functional images were co-registered to high-resolution structure T1 images. Functional images were normalized to a common space (Montreal Neurological Institute (MNI) space). The spatial smoothing was conducted with 4-mm full width half maximum (FWHM) kernel (see Ashburner, et al.^14^ for details of preprocessing for fMRI).

For fMRI analysis based GLM, we constructed three separate design matrices for contrast analyses and parametric modulation analysis. In first design matrix, five regressors were used as follows: (${a}_{1}$) onset of the presentation of S2 in brand extension stimuli leading to positive responses (*Positive*); (${b}_{1}$) onset of the presentation of S2 in brand extension stimuli leading to negative responses (*Negative*); (${c}_{1}$) onset of the presentation of S1 in all brand extension stimuli (*S1*); (${d}_{1}$) onset of the fixation before presenting all brand extension stimuli (*Fixation*); and $({e}_{1}$) onset of participant’s response (i.e., button press) to all brand extension stimuli (*Response*). Additionally, we put six head motion parameters (i.e., x, y, z, pitch, roll, and yaw) as nuisance regressors in the design matrix.

In the second design matrix, we constructed two regressors as follows; $({a}_{2}$) onset of the presentation of S2 in beverage-beverage brand extension stimuli (*BB*); (${b}_{2}$) onset of the presentation of S2 in beverage-household appliance brand extension stimuli (*BH*); (${c}_{2}$) onset of participant’s response (i.e., button press) to all brand extension stimuli (*Response*). Nuisance regressors were added as same as the first design matrix.

From the first and second design matrix, we constructed eight contrast models as follows: (1) *Positive* > *Negative responses* in S2 (i.e., ${a}_{1}-{b}_{1}$ above); (2) *Negative* > *Positive responses* in S2 (i.e., ${b}_{1}-{a}_{1}$ above); (3) *Evaluation* (at the presentation of S2) > *Rest* (at the fixation) (i.e., ${a}_{1}+{b}_{1}-{d}_{1}$ above); (4) *Rest* (at the fixation) > *Evaluation* (at the presentation of S2) (i.e., ${d}_{1}-{a}_{1}+{b}_{1}$ above); (5) *Stimulus* (at the presentation of either S1 or S2) > *Rest* (at the fixation) (i.e., ${a}_{1}+{b}_{1}+{c}_{1}-{d}_{1}$ above); (6) *Rest* (at the fixation) > *Stimulus* (at the presentation of either S1 or S2) (i.e., ${d}_{1}-{a}_{1}+{b}_{1}+{c}_{1}$ above); (7) *BB* > *BH* in S2 (i.e., ${a}_{2}-{b}_{2}$ above); and (8) *BH* > *BB* in S2 (i.e., ${b}_{2}-{a}_{2}$ above). Note that the onset of participant’s response regressors (${e}_{1}$ and ${c}_{2}$) was used as to reduce the effect of hand movement when participants responded. Contrast models 1 and 2 were designed to find neural activity modulated by the valence of emotion (or acceptance), either positively (model 1) or negatively (model 2). Contrast models 3–6 were designed to find neural activity modulated by the evaluation process, and contrast model 7 and 8 were designed to validate the effect of emotion.

Finally, we constructed the last design matrix with four regressors as follows; (${a}_{3}$) onset of the presentation of S2 in all brand extension stimuli (*S2*); (${b}_{3}$) onset of the fixation before presenting all brand extension stimuli (*Fixation*); and (${c}_{3}$) onset of participant’s response (i.e., button press) to all brand extension stimuli (*Response*). In addition, we put two parametric modulators; (${d}_{3}$) response value; and (${e}_{3}$) absolute value of response. Six nuisance regressors were added to the last design matrix. Using this design matrix, we conducted three parametric modulation analyses as follows: (1) *Positive tracking* (i.e., ${d}_{3}-{b}_{3}$ above); (2) *Negative tracking* (and i.e., ${b}_{3}-{d}_{3}$ above); and (3) *Absolute tracking* (i.e., ${e}_{3}-{b}_{3}$ above).

Note that, for all contrast and parametric modulation analyses, the sum of contrast weights both before and after negative sign ($-$) was 1, resulting in a total sum of contrast weights as 0. In the first level analysis, contrast images were generated for each participant with detrending process. Then, the second-level analysis was conducted by computing a one-sample t-test with individual contrast images obtained from the first-level analysis. Family-wise error (FWE) was used for multiple comparisons. We identified only regions where cluster size is more than 10. Finally, we labelled anatomical locations of significant clusters using the automated anatomical labeling (AAL) toolbox^15^.

**Figure 2:**
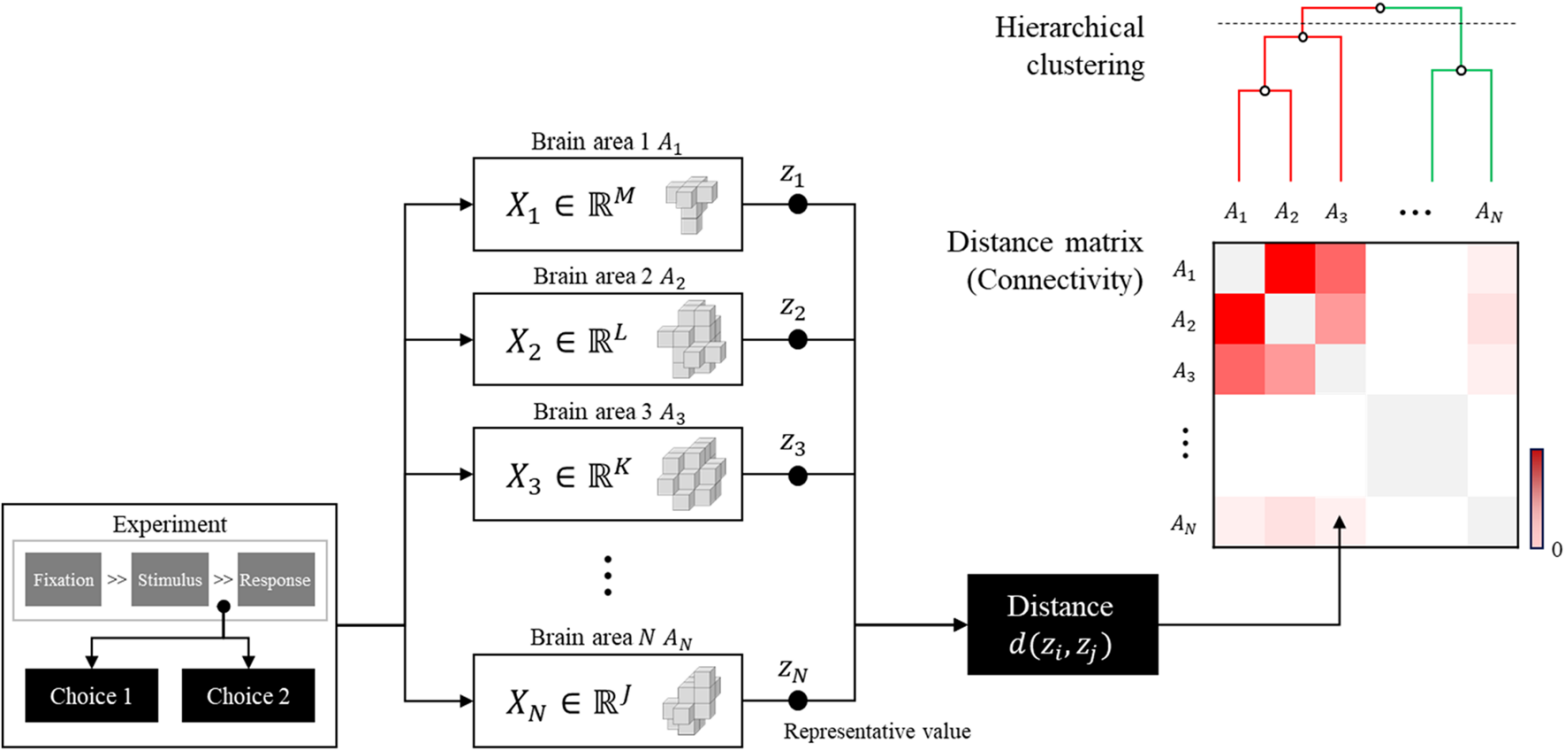
Hierarchical clustering to partition the brain activity pattern during the experimental task. Suppose there are ${\varvec{N}}$ brain regions of interests. We can extract brain activation patterns ${{\varvec{X}}}_{{\varvec{i}}}$ during evaluation process for each brain regions. Because each brain regions ${{\varvec{A}}}_{{\varvec{i}}}$ has its own shape and voxel size, however, the voxel intensity itself cannot be compared between brain regions. Hence, representative values (e.g., mean intensity or eigenimage) ${{\varvec{z}}}_{{\varvec{i}}}$ for each sample and brain regions were extracted. The distance (or dissimilarity) ${\varvec{d}}({{\varvec{z}}}_{{\varvec{i}}},{{\varvec{z}}}_{{\varvec{j}}})$ between each brain regions to create a distance matrix. Hierarchical clustering can then be performed using a distance matrix. Note that Pearson’s correlation distance was used as a distance metric in our analysis.

Because diverse brain areas activated during brand extension evaluation (see “Results”), we additionally conducted a hierarchical clustering analysis to dissect these activations. Hierarchical clustering is an agglomerative clustering method grouping data (i.e., ROIs) according to the informational distance between them. In fMRI, it has been used to partition brain areas based on fMRI connectivity data^16–19^ or to conduct fMRI parcellation study^20^. In the present study, we used hierarchical clustering to partition regional brain activities during brand extension evaluation (Fig. 2). The data for the hierarchical clustering analysis were aggregated on a per-trial basis. In other words, we extracted 180 data samples from each of the 180 trials in each participant, resulting in a total of 4860 samples. Then, we excluded 53 samples where the behavioral response was missed (i.e., participants failed to respond within 3 s) and used 4807 samples for the hierarchical clustering analysis. For each sample, we extracted trial-by-trial beta images using GLM. Then, we determined region of interest (ROIs) that included significant clusters observed in the second level analysis above. From each ROI, we extracted beta values and took the first eigenimage from each ROI mask to represent all the voxels within the ROI. Because we did not fix time for response, we assumed that the first TR image acquired after S2 onset would represent cognitive processes related to brand extension evaluation. For the hierarchical clustering analysis, we calculated pairwise Pearson’s correlation distances between brain activities between all the ROIs and carried out a complete-linkage hierarchical clustering using the R package called ComplexHeatmap (ver. 2.2.0)^21,22^.

## Results

**Figure 3:**
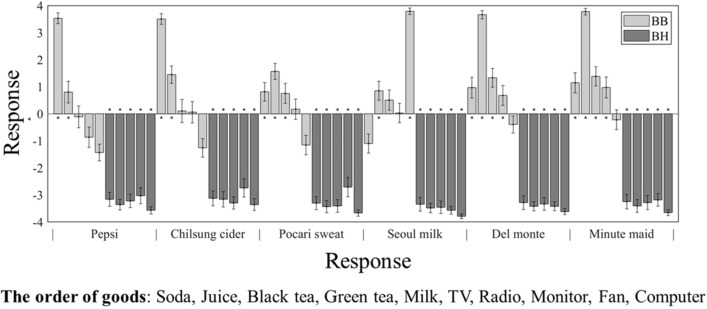
Mean response of acceptability toward each brand extension pair. light gray and dark gray box indicate beverage-to-beverage (*BB*) and beverage-to-household appliance (*BH*) brand extension pair stimuli (pre-determined by experimenters) each. Asterisk indicates that response to that pair were significantly far from zero (i.e., either positive or negative for *BB* and *BH* each) (*ps* < 0.05). All light gray boxes were expected to show significantly high fit response (asterisk, *ps* < 0.05). However, there was exceptions (vacant, *ps* ≥ 0.05).

**Figure 4:**
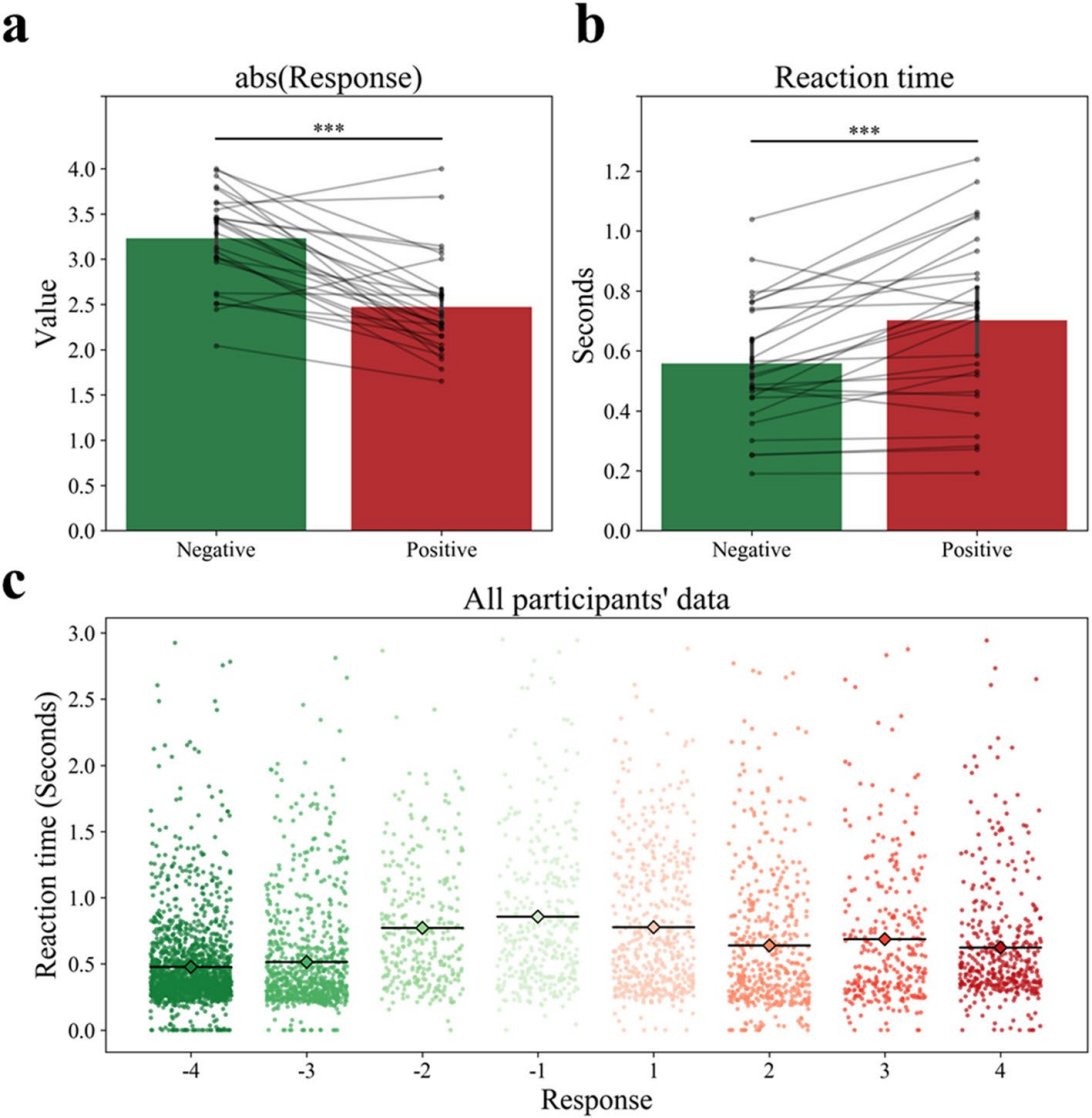
Comparison of positive and negative responses with respect to (**a**) absolute values of response and (**b**,**c**) reaction time. Black lines indicate each participant’s mean response value (a total of 27 participants; see in the text how response value was determined). An asterisk indicates a significant difference between two groups (*ps* < 0.001). (**a**) Higher absolute response values represent that participants either more strongly accept (*Positive*) or deny (*Negative*) a given brand extension sample. (**b**) The reaction time (RT) was measured in second. (**c**) All participants’ data was aggregated and plotted with random jittering. Diamond with black line indicate mean of reaction time for each response group.

The first behavioral analysis comparing mean response toward each stimulus showed noticeable result. Contrary to our expectation that participant will respond to *BB* stimuli positively, one-sided t-tests showed that some of the response to *BB* were not significantly positive while all the responses to *BH* were significantly negative (Fig. 3). This result affected the further analyses. The paired t-test revealed that the mean absolute value of negative responses (M 3.23, SE 1.00) was significantly larger than that of positive responses (M 2.47, SE 0.11) (t(26) = − 6.3378, *p* < 0.001) (Fig. 4a). It also showed that RT of negative responses (M 0.56, SE 0.040) was significantly faster than that of positive responses (M 0.70, SE 0.055) (t(26) = 4.6898, *p* < 0.001) (Fig. 4b). Lastly, Fig. 4c showed the distribution of relatively faster RT for extreme responses than non-extreme responses and negative than positive responses.

**Figure 5:**
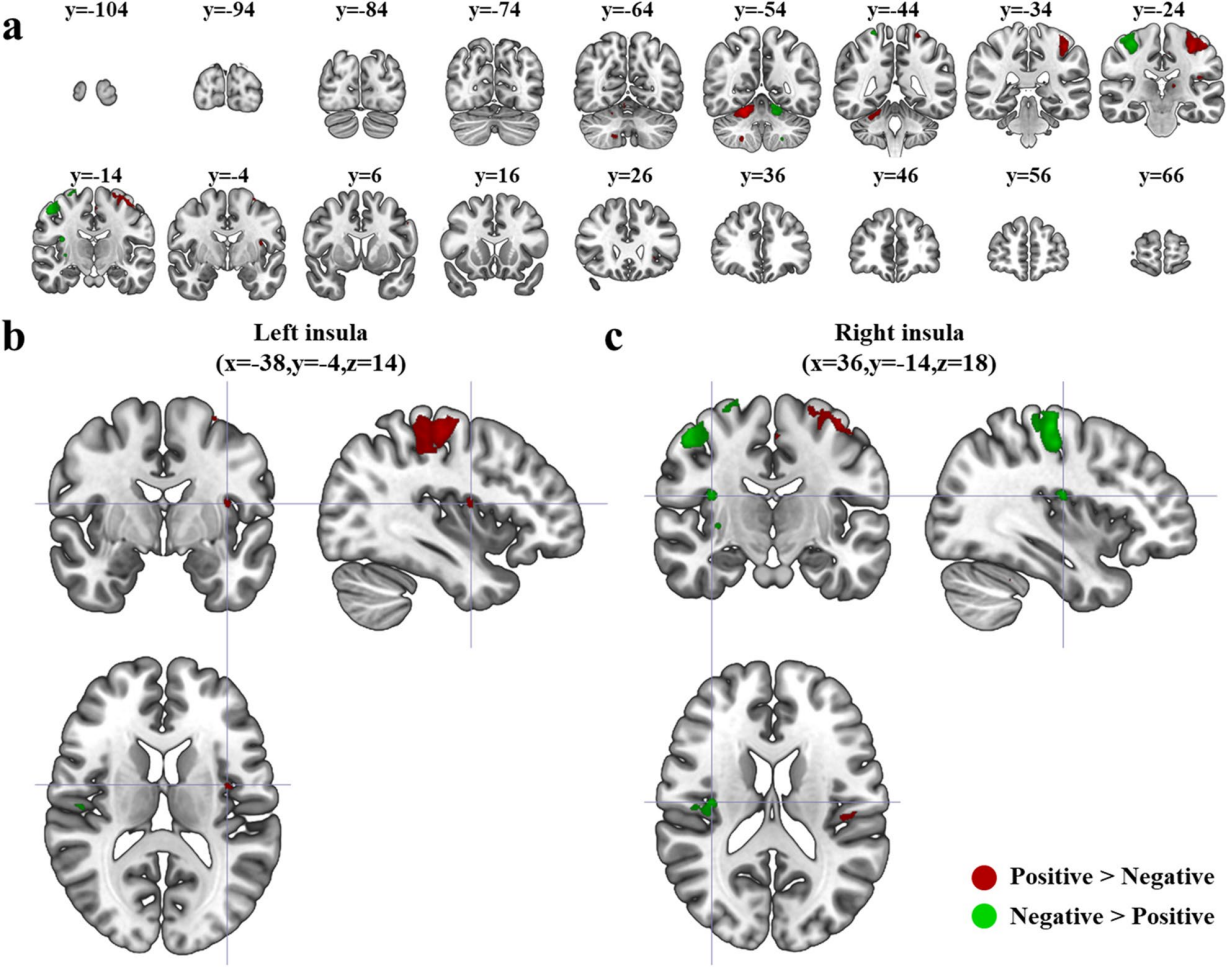
Brain regions revealing significant activations with the positive or negative evaluation of brand extension. (**a**) Twenty slices of the coronal view showing significant activations in either *Positive* > *Negative* or *Negative* > *Positive* contrast (FWE corrected, *p* < 0.05, cluster size > 10). Bilateral activations are observed in cerebellum, precentral gyri, postcentral gyri, insula, and putamen; (**b**) Left insula shows significant activation in *Positive* > *Negative* contrast (FWE corrected, *p* < 0.05, cluster size > 10) and (**c**) Right insula shows significant activation in *Negative* > *Positive* contrast (FWE corrected, *p* < 0.05, cluster size > 10).

We observed significant BOLD responses in various brain regions by contrasting response conditions using GLM analysis. The whole brain analysis of contrast model 1 (*Positive* > *Negative*) identified several clusters of voxels with significant BOLD responses in: right cerebellum 4/5, right cerebellum 6, right cerebellum 8, left inferior parietal gyrus, left insula, left postcentral gyrus, left postcentral gyrus, left precentral gyrus, left putamen, left rolandic operculum, left supplementary motor area, and left thalamus (Table 1). On the other hand, contrast model 2 (*Negative* > *Positive responses*) found significant clusters in: left cerebellum 4/5, left cerebellum 8, right insula, right postcentral gyrus, right precentral gyrus, and right putamen (Table 1). Notably, these two sets of brain regions with opposite contrasts were virtually symmetrical (Fig. 5a). These contrast analyses also unveiled the lateralized activation of insula. *Positive* > *Negative* revealed significant activation in left insula (Fig. 5b), while *Negative* > *Positive* revealed significant activation in right insula (Fig. 5c).

**Table 1:** MNI coordinates of results for contrast model 1 and 2.

Brain regions	Side	Cluster size	MNI coordinate			T	Z	Brain regions	Side	Cluster size	MNI coordinate			T	Z
			X	Y	Z						X	Y	Z		
[Contrast model 1] *Positive response* > *Negative response*								[Contrast model 2] *Negative response* > *Positive response*							
Cerebellum 4/5	R	358	18	− 52	− 18	14.43	7.50	Cerebellum 4/5	L	145	− 16	− 52	− 20	12.29	7.01
Cerebellum 6	R	17	18	− 68	− 20	8.93	5.99								
Cerebellum 8	R	104	20	− 58	− 52	9.76	6.27	Cerebellum 8	L	12	− 24	− 52	− 52	7.95	5.61
Inferior parietal gyrus	L	11	− 44	− 28	38	7.47	5.41								
Insula	L	10	− 38	− 4	14	7.73	5.52	Insula	R	60	36	− 14	18	10.55	6.52
Postcentral gyrus	L	1000	− 34	− 24	52	12.56	7.07	Postcentral gyrus	R	42	24	− 42	70	10.38	6.47
Postcentral gyrus	L	45	− 24	− 40	66	8.25	5.73								
Precentral gyrus	L	48	− 48	10	30	9.06	6.03	Precentral gyrus	R	665	38	− 20	60	14.08	7.43
Putamen	L	18	− 30	− 6	− 2	7.24	5.31	Putamen	R	10	34	− 12	0	8.85	5.96
Rolandic operculum	L	18	− 42	− 24	20	7.62	5.48								
Supplementary motor area	L	13	− 2	− 14	54	7.55	5.45								

Row orders was sorted in alphabetical order with a few vacant rows to maximally visualize difference of two contrast models.

No significant activation was observed from both contrast model 3 (*Evaluation* > *Rest*) and model 4 (*Rest* > *Evaluation*). On the other hand, contrast model 5 (*Stimulus* > *Rest*) presented significant clusters in left inferior occipital gyrus, left lingual gyrus, right lingual gyrus, and left middle occipital gyrus (Table 2). On the contrary, contrast model 6 (*Rest* > *Stimulus*) showed more brain activation as follows: left cerebellum 8, left cuneus, right inferior parietal gyrus, left middle frontal gyrus, right superior parietal gyrus, and right supramarginal gyrus (Table 2).

**Table 2:** MNI coordinates of results for contrast model 3, 4, 5, and 6.

Brain regions	Side	Cluster size	MNI coordinate			T	Z	Brain regions	Side	Cluster size	MNI coordinate			T	Z
			X	Y	Z						X	Y	Z		
[Contrast model 3] *Evaluation* > *Rest*								[Contrast model 4] *Rest* > *Evaluation*							
No activated cluster was found								No activated cluster was found							
[Contrast model 5] *Stimulus* > *Rest*								[Contrast model 6] *Rest* > *Stimulus*							
								Cerebellum 8	L	10	− 8	− 66	− 50	8.14	5.69
								Cuneus	L	34	− 6	− 92	18	8.97	6.00
Inferior occipital gyrus	L	11	− 40	− 74	− 12	7.89	5.59	Inferior parietal gyrus	R	17	52	− 44	56	8.30	5.75
Lingual gyrus	L	40	− 18	− 86	− 14	8.43	5.80								
Lingual gyrus	R	60	20	− 90	− 6	8.33	5.76								
								Middle frontal gyrus	L	11	− 42	22	46	9.47	6.18
Middle occipital gyrus	L	33	− 28	− 92	4	7.63	5.48								
								Superior parietal gyrus	R	12	18	− 54	72	7.29	5.33
								Supramarginal gyrus	R	64	62	− 30	42	9.42	6.16

Row orders was sorted in alphabetical order with a few vacant rows to maximally visualize difference of two contrast models.

Table 3 shows the result of second design matrix. Contrast model 7 (*BB* > *BH*) showed significant activation in right cerebellum 4/5, left cerebellum 6, right cerebellum 8, left postcentral gyrus, left precentral gyrus, left precentral gyrus, and left thalamus. In contrast, contrast model 8 (*BH* > *BB*) showed brain areas’ activation as follows: left cerebellum 4/5, right precentral gyrus, and right precentral gyrus.

**Table 3:** MNI coordinates of results for contrast model 7 and 8.

Brain regions	Side	Cluster size	MNI coordinate			T	Z	Brain regions	Side	Cluster size	MNI coordinate			T	Z
			X	Y	Z						X	Y	Z		
[Contrast model 7] *BB* > *BH*								[Contrast model 8] *BH* > *BB*							
Cerebellum 4/5	R	80	16	− 52	− 18	9.39	6.15	Cerebellum 4/5	L	52	− 20	− 48	− 24	8.08	5.67
Cerebellum 6	L	12	− 24	− 68	− 26	8.40	5.79								
Cerebellum 8	R	30	18	− 60	− 46	9.21	6.09								
Postcentral gyrus	L	300	− 42	− 28	50	10.20	6.42								
Precentral gyrus	L	41	− 50	12	32	8.29	5.75	Precentral gyrus	R	198	36	− 22	60	10.86	6.62
Precentral gyrus	L	10	− 26	− 12	66	7.48	5.42	Precentral gyrus	R	17	48	− 10	52	7.87	5.58
Thalamus	L	16	− 16	− 26	12	7.74	5.53								

Row orders was sorted in alphabetical order with a few vacant rows to maximally visualize difference of two contrast models.

Finally, Table 4 shows the result of parametric modulation model 1 and 3. In parametric modulation model 2 (*Negative tracking*), there was no significantly activated brain regions. In parametric modulation model 1 (*Positive tracking*) showed significant activation in right cerebellum 4/5, right cerebellum 6, left fusiform gyrus, left fusiform gyrus, right fusiform gyrus, right inferior frontal gyrus, left insula, right insula, right precuneus, right precuneus, right superior temporal gyrus, and left thalamus. On the other hand, parametric modulation model 3 (*Absolute tracking*) showed significant activations in left fusiform gyrus, left fusiform gyrus, right fusiform gyrus, left lingual gyrus, right middle temporal gyrus, right middle temporal gyrus, and right superior temporal gyrus.

**Table 4:** MNI coordinates of results for parametric modulation model 1 and 3.

Brain regions	Side	Cluster size	MNI coordinate			T	Z	Brain regions	Side	Cluster size	MNI coordinate			T	Z
			X	Y	Z						X	Y	Z		
[Parametric modulation model 1] *Positive tracking*								[Parametric modulation model 3] *Absolute tracking*							
Cerebellum 4/5	R	10	20	− 52	− 20	8.04	5.65								
Cerebellum 6	R	21	30	− 52	− 22	8.15	5.69								
Fusiform gyrus	L	22	− 36	− 60	− 18	9.00	6.01	Fusiform gyrus	L	51	− 34	− 42	− 22	9.76	6.28
Fusiform gyrus	L	33	− 40	− 48	− 20	8.69	5.90	Fusiform gyrus	L	73	− 34	− 60	− 16	9.44	6.17
Fusiform gyrus	R	10	38	− 62	− 16	7.19	5.29	Fusiform gyrus	R	54	34	− 54	− 16	7.89	5.59
Inferior frontal gyrus	R	26	50	22	28	8.64	5.88								
Insula	L	38	− 30	26	4	9.94	6.33								
Insula	R	49	34	18	8	9.11	6.05								
								Lingual gyrus	L	12	− 28	− 80	− 14	8.97	6.00
								Middle temporal gyrus	R	11	50	− 22	− 8	8.32	5.76
								Middle temporal gyrus	R	18	54	0	− 18	7.96	5.62
Precuneus	R	17	22	− 60	28	8.78	5.93								
Precuneus	R	27	16	− 54	22	8.58	5.86								
Superior temporal gyrus	R	12	56	− 44	18	9.02	6.02								
Thalamus	L	23	− 2	− 30	− 2	9.02	6.02								

There was no significantly activated regions in parametric modulation 2 (*Negative tracking*). Row orders was sorted in alphabetical order with a few vacant rows to maximally visualize difference of two contrast models.

**Figure 6:**
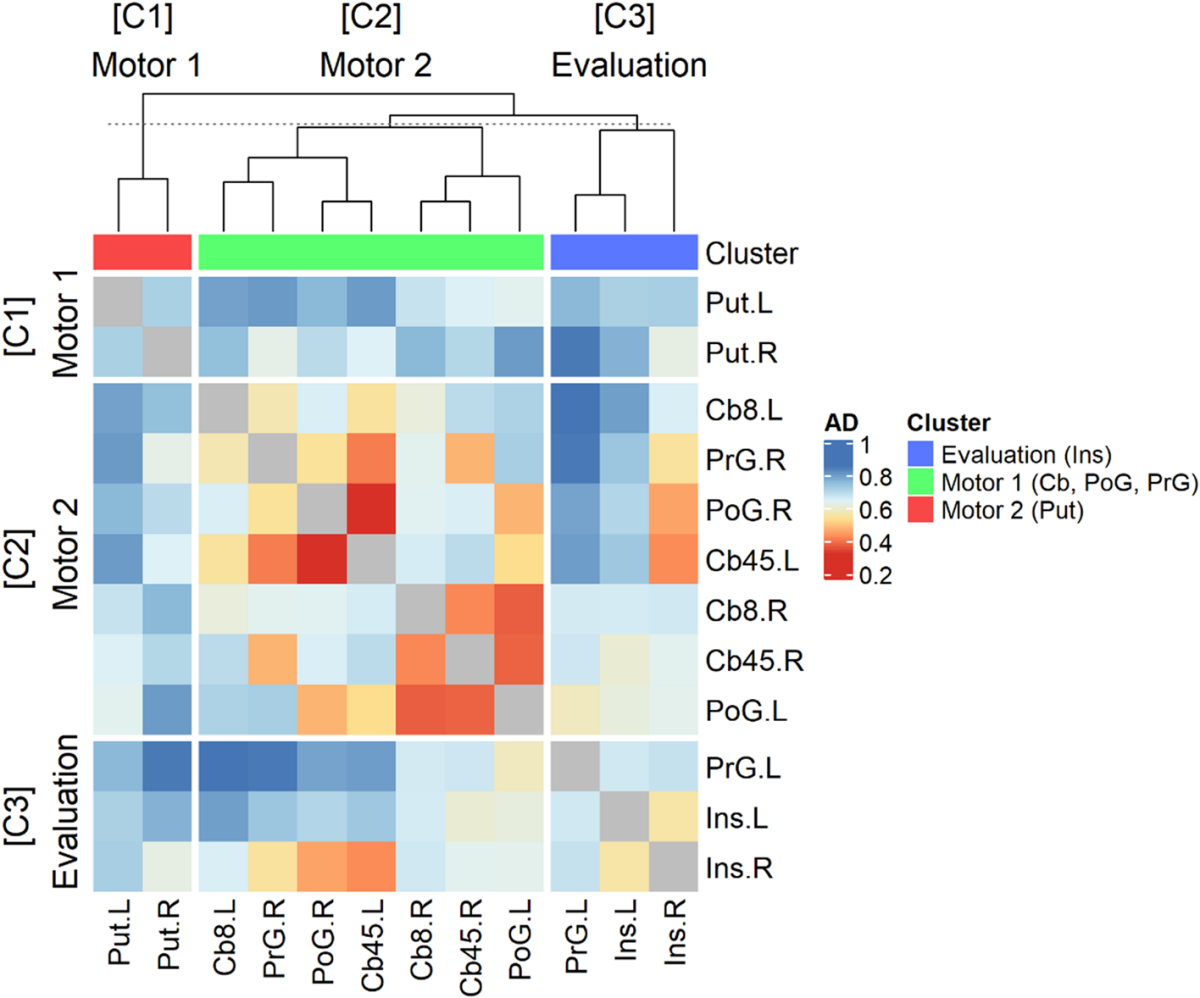
A heatmap with the result of hierarchical clustering. The order of both row and column was determined by hierarchical clustering algorithm as follows: (1) left putamen (Put.L), (2) right putamen (Put.R), (3) left cerebellum 8 (Cb8.L), (4) left cerebellum 4/5 (Cb45.L), (5) right cerebellum 8 (Cb8.R), (6) right cerebellum 4/5 (Cb45.R), (7) right precentral gyrus (PrG.R), (8) left postcentral gyrus (PoG.L), (9) right postcentral gyrus (PoG.R), (10) left precentral gyrus (PrG.L), (11) left insula cortex (Ins.L), and (12) right insula cortex (Ins.R). Each cell indicates Pearson correlation distance between brain regions.

As a connectivity analysis, we clustered BOLD responses in twelve activated brain regions which are symmetrically activated in the contrast model 1 (*Positive* > *Negative*) and 2 (*Negative* > *Positive*): left/right cerebellum, left/right insula, left/right postcentral gyrus, left/right precentral gyrus, and left/right putamen. The hierarchical clustering analysis divided these regions into three clusters (Fig. 6). The first cluster consisted of both left and right putamen. The second cluster included sensorimotor areas such as bilateral cerebellum, bilateral postcentral gyrus, and right precentral gyrus. The third cluster included bilateral insula and left precentral gyrus.

## Discussion

Given that brand extension is increasingly used as a strategy for many companies, the question of how consumers assess new brand extension has drawn constant attention from consumer researchers and neuroscientists. The current study adds to this growing body of literature by investigating the involvement of in-depth brain areas during brain extension evaluation process.

Previous EEG studies suggest that the main cognitive process operating for brand extension evaluation is categorization^4^ or conflict mismatch^5^ between parent brand and extended goods. Among them, only one study considered the impact of emotion on brand extension evaluation^13^. However, that study used emotional stimuli as a primer prior to the brand extension evaluation in which the emotion was considered as an exogenous effect. In contrast, the present study speculates a possibility of emotional involvement in brand extension evaluation, shown as insular activity.

The behavioral result of the current study shows that the absolute response value was larger and reaction time was faster in the negative responses (Fig. 4b). Besides, the results also revealed evaluating obvious match/mismatch (e.g., Pepsi-soda or Pepsi-computer) may be much easier than nuanced mismatch (Pepsi-juice) because reaction time for extreme stimulus was shorter than non-extreme stimulus ones (Fig. 4c). This can be supported by fMRI analyses by contrasting *NegBB & PosBH* > *PosBB & NegBH*, and vice versa. However, in the present study, samples of either *NegBB* or *PosBH* were not enough to conduct GLM contrast analysis. Therefore, we will address this in a future study.

In the GLM analyses, the most noticeable area during brand extension evaluation (*Positive* > *Negative*, and vice versa) was insula, although there were other high significant regions such as cerebellum or pre/postcentral gyri (Table 1). However, interestingly, parametric modulation model 1 (*Positive tracking*) showed that brain activity in bilateral insula was shown to best explain subjects' responses with the highest T values (Table 4). On the other hand, insular activation pattern was not presented in other parametric modulation models (*Negative tracking* and *Absolute tracking*). Because *Absolute tracking* model did not show insula activity, we speculate the activity of insula is related to the valence level.

Another key finding of the present study is the lateralized activation of insula cortex according to emotional (positive and negative) brand extension evaluation. We observed that the left and right insula cortices was activated for positive and negative brand extension evaluation, respectively (Fig. 5b,c). Insula cortex is known for the integration of interoceptive information and emotional experiences^23,24^. In addition, other studies revealed that emotional processing in the insula is lateralized based on the stimulus valence (positive/negative emotions) and behavior (approach/withdrawal)^23,25,26^. In particular, the stimulation of the left insula resulted in changes in parasympathetic functions involving positive affect and approach behavior, whereas the right insula has been involved in the top-down control of sympathetic-nervous system functioning associated with negative affect and avoidance behavior^25^. Craig^23^ conducted a meta-analysis to emphasize the importance of right insula in emotional processing and Critchley et al.^26^ added to empirical evidence by experimentally showing a close relationship between right insula and unpleasant emotions. Accordingly, the lateralized activation of the insula found in this study suggests that emotional processes as well as cognitive processes (e.g., categorization theory) may be involved in brand extension evaluation.

Our contrast analyses also revealed the lateralized activations according to behavioral responses in the brain regions related to sensorimotor functions, including putamen, cerebellum, precentral, and postcentral gyri (Fig. 5a). In line with previous reports^27,28^ these brain regions were activated contralateral to finger movements such that right finger movements during positive responses activated left putamen, precentral and postcentral gyri, and cerebellum whereas left finger movements during negative responses activated right putamen, precentral and postcentral gyri and cerebellum. Since our experimental design let participants express positive evaluation with the right fingers and negative one with the left fingers, *Positive* > *Negative* and *Negative* > *Positive* contrast analyses used in this study inevitably exhibited activations in brain regions related to sensorimotor functions. Nonetheless, we speculate that insular activities are not result of sensorimotor response due to two reasons. First, brain activities of those sensorimotor regions explained behavioral response not well (Table 4). On the other hand, insular activity could explain behavioral response well. Besides, additional hierarchical clustering analysis (Fig. 6) revealed that activations in these sensorimotor areas are separately working with that of insula. If brain activities of insula and sensorimotor areas are coupled, they would be grouped into the sample cluster after hierarchical clustering.

There is a chance that other alternative cognitive processes (i.e., error awareness^29^ and salience processing) might be engaged in a match-mismatch judgment task designed in this study, modulating insula activity. However, our experimental task involved text-based semantic evaluation, rather than error awareness, because participants were asked to respond subjectively with no feedback of “correctness/incorrectness” about their response. Furthermore, we did not give participants any feedback about the consistency of their response across sessions. Therefore, we did not consider error awareness processing as a main process underlying the brand extension evaluation task.

In addition, visual properties (e.g., color, font or font size) were no different among the stimuli, ruling out a possibility of the effect of visual saliency on positive and negative evaluation. On the other hand, preference might be one of the alternative explanations. However, if participants preferred or did not prefer one of the brands or goods, it might not only evoke faster recognition and but also more positive/negative “emotions” regarding that stimulus. It means that preference saliency processing could be involved in our task, which also induce emotional processing. Thus, it could be a part of our account of emotion processing during brand extension evaluation.

Another plausible account is an “expectation-mismatch processing”. When participants faced a parent brand (S1), they might expect that the following extended goods (S2) was similar to the main category of S1. But, if S2 was different from S1’s main category, mismatch processing could occur. However, there is no neuroscientific evidence showing that the insula activity represents mismatch processing, to the best of our knowledge.

On the other hand, it is relatively more evident that emotional processing could account for neural activity observed in this study. This conjecture is based on the following results: (1) Insula was activated showing ` other regions. (4) Insular activity was separated from sensorimotor responses in clustering analysis. Because previous studies also described that insula reflects the integration of interoceptive information^23,24^ and that its activity is lateralized to affective levels of stimulus or behavior^23,25,26^, we guess that the insula activity observed in this study can be elucidated by the emotional processing of affective levels of a given brand extension stimulus, induced by integration of interoceptive evaluation of it. Although we agree that there can be other possible accounts for insula activity during brand extension evaluation, we conclude that emotion processing is one of the most plausible underlying processes at this initial stage of neuroimaging studies on brand extension.

There was no brain region showing significant activation in the contrast between *Evaluation* > *Rest,* and vice versa (Table 2). However, in a further analysis where we eliminated a threshold of the minimum fMRI voxel cluster size from the *Evaluation vs Rest* contrast, we observed significant activations in the left occipital cortex. Although the cluster size was only either two (x = − 34, y = − 92, and z = 0; FWE corrected, *p* < 0.05) or three (x = − 26, y = − 96, and z = 2; FWE corrected, *p* < 0.05), this result may indicate visual processing during brand extension evaluation. Similarly, the contrast of *Stimulus* > *Rest* revealed activations in the lingual and occipital gyri during the visual presentation of a pair of S1 and S2. These brain regions are linked to processing visual information, especially related to letters^30^. Therefore, we suspect that the presentation of the parent brand and extended goods names in letters might evoke active processing in these regions. In addition, default mode network (DMN)-related brain regions, including cuneus, inferior parietal gyrus, and middle frontal gyrus, were activated in *Rest* > *Stimulus.* It indicates that participants were actively engaged in brand extension evaluation during the task.

As a first attempt to investigate neural substrates of brand extension evaluation using fMRI, we shed light on the involvement of in-depth brain areas related to emotional processing in the evaluation. However, the present study has its own limitations as well. First, we suggested a new functional connectivity analysis method using hierarchical clustering (Fig. 2). some previous methods, such as the representational similarity analysis (RSA)^31^, analyze connectivity based on a pre-defined stimulus set. For instance, RSA divides the dataset according to the stimulus and calculates the distance between brain responses for each stimulus to create a distance matrix. So, RSA needed to segment the dataset into subsets for each stimulus, reducing the number of samples and thus weakening stability of the distance measurement. RSA is also not suitable for analyzing behavior-based connectivity because it cannot generate a distance matrix when some participants do not choose a specific response at all, which is the case of the present study. Therefore, we devised a hierarchical cluster analysis method that inspects brain connectivity based on behavioral responses. This method can create a distance matrix using the entire dataset and become more suitable for our analysis purpose. Although our method has some advantages and the present study shows a plausible result, it is necessary to verify whether this method is task independent, which will be addressed in a future study. Besides, we conducted the hierarchical clustering by concatenating all participants’ data to take advantage of a larger number of samples. As a result, the statistical evaluation was difficult to apply in this analysis, which might limit the interpretation of current clustering analysis results. Second, *Evaluation vs. Rest* contrast was conducted to estimate the effect of the evaluation process on brain activity. However, there was no significant activation in the contrast between *Evaluation vs. Rest* found in this study. We assume that this is partly due to the design of the experiment, where sham text stimuli should also be included to contrast *Evaluation* versus *Control*, instead of *Rest*. In follow-up studies, we will address this shortcoming of experimental design. Finally, we adopted S1–S2 paradigm which has been used as an experimental paradigm for brand extension evaluation in EEG studies^4,6,7^. However, there was no study to verify the experimental paradigm. Thus, in the future study, there need control condition to show such evaluative processing is specific to brand extension.

A number of studies have been conducted since 1990 when Aaker and Keller^2^ first studied the consumer evaluation process for brand extension, but the process of endogenous appraisal of brand extension evaluation has yet to be studied. Few studies have examined the exogenous effects of emotions on brand extension^13,32^. Therefore, despite some limitations, the present study may contribute to brand extension research as the first study to present the endogenous effect of emotion on brand extension assessment. Furthermore, our results showed a quick and clear pattern of decision on brand extension that felt negative. The results presented in this study together may provide marketing researchers useful clues to understand consumer assessment of brand extension. For instance, when asked to evaluate brand expansion, consumers’ response time may provide a fast and efficient measurement without the need for complex experiments or apparatus. Also, while human information processing model of brand extension is not yet clear, it should be underlined that consumers can evaluate brand extension on an emotional basis.

## Conclusions

To conclude, the present study explored brain regions over the whole involved in brand extension assessment. The fMRI technique was used to investigate neural responses when participants evaluated their acceptability for a pair of parent brand and its new extension. From the behavioral result, we observed that participants evaluated brand extension more quickly and decisively when they judged it negatively. The GLM-based fMRI analyses revealed the lateralized activation of the insula cortex in a way that positive and negative evaluation activated the left and right insula, respectively. Besides, parametric GLM models showed insular activity explain behavioral response well. Finally, a follow-up clustering analysis showed that these insula activations were separated from those in brain regions related to sensorimotor processing. Based on primary functions of the insula cortex regarding emotional experiences, our results may suggest that emotional process precedes cognitive one in brand extension evaluation task.

## Data Availability

The datasets generated during and/or analyzed during the current study are available from the corresponding author on reasonable request.
